# Robust Prediction of Cardiorespiratory Signals from a Multimodal Physiological System on the Upper Arm

**DOI:** 10.3390/bios15080493

**Published:** 2025-08-01

**Authors:** Kimberly L. Branan, Rachel Kurian, Justin P. McMurray, Madhav Erraguntla, Ricardo Gutierrez-Osuna, Gerard L. Coté

**Affiliations:** 1 Department of Biomedical Engineering, Texas A&M University, College Station, TX 77843, USA; 2 Department of Electrical and Computer Engineering, Texas A&M University, College Station, TX 77843, USA; 3 Department of Industrial and Systems Engineering, Texas A&M University, College Station, TX 77843, USA; 4 Department of Computer Science and Engineering, Texas A&M University, College Station, TX 77843, USA; 5 Texas A&M Engineering Experiment Station, Center for Remote Health Technologies and Systems, College Station, TX 77843, USA

**Keywords:** bioimpedance, electrocardiography, photoplethysmography, heart rate, respiration rate, wireless health monitoring, noise artifacts, multimodal wearable

## Abstract

Many commercial wearable sensor systems typically rely on a single continuous cardiorespiratory sensing modality, photoplethysmography (PPG), which suffers from inherent biases (i.e., differences in skin tone) and noise (e.g., motion and pressure artifacts). In this research, we present a wearable device that provides robust estimates of cardiorespiratory variables by combining three physiological signals from the upper arm: multiwavelength PPG, single-sided electrocardiography (SS-ECG), and bioimpedance plethysmography (BioZ), along with an inertial measurement unit (IMU) providing 3-axis accelerometry and gyroscope information. We evaluated the multimodal device on 16 subjects by its ability to estimate heart rate (HR) and breathing rate (BR) in the presence of various static and dynamic noise sources (e.g., skin tone and motion). We proposed a hierarchical approach that considers the subject’s skin tone and signal quality to select the optimal sensing modality for estimating HR and BR. Our results indicate that, when estimating HR, there is a trade-off between accuracy and robustness, with SS-ECG providing the highest accuracy (low mean absolute error; MAE) but low reliability (higher rates of sensor failure), and PPG/BioZ having lower accuracy but higher reliability. When estimating BR, we find that fusing estimates from multiple modalities via ensemble bagged tree regression outperforms single-modality estimates. These results indicate that multimodal approaches to cardiorespiratory monitoring can overcome the accuracy–robustness trade-off that occurs when using single-modality approaches.

## 1. Introduction

Wearable technology has opened the door to continuous and remote health monitoring. With advances in technology (e.g., miniaturization), devices can now contain multiple sensing modalities to monitor the cardiac state of a patient, such as photoplethysmography (PPG), bioimpedance plethysmography (BioZ), and electrocardiography (ECG). The main challenge for wearable technology to transition to remote health monitoring applications is noise that emanates from the subject being monitored, since instrument noise, such as thermal noise or flicker noise (e.g., 1/f noise) are typically quite low in most commercial systems. Thus, for the purpose of this study, we define noise as any interference from the subject under test that results in a misrepresentation of the target physiological signal. Physiological signals from wearable devices are prone to noise given their relatively low amplitude (when sampled noninvasively) and the chaotic ambulatory environments in which the signals are measured. Therefore, for wearable sensors to transition to health applications, it is critical to identify how such noise sources affect various physiological signals and how they might be mitigated.

For this study, we classify noise into four categories based on its source: physiological (e.g., involuntary muscle contraction, respiration, skin tone), external (e.g., motion), hardware (e.g., electromagnetic interference, crosstalk), and human–device interface (e.g., electrode–skin interface, device location) [1,2,3,4]. Since the effect of each type of noise on signal quality is modality-dependent, we examined how different noise sources affect the accuracy of PPG, BioZ, and single-sided ECG (SS-ECG) from the upper arm when estimating heart rate (HR) and breathing rate (BR). Though these three modalities have previously been used to monitor HR and BR independently [5,6,7], to our knowledge no device combines all three modalities to jointly estimate HR and BR.

The three sensing modalities provide independent measurements of cardiac activity. PPG is an optical measurement of changes in the blood volume of the probed tissue and produces an oscillatory signal that matches the timing of individual heartbeats [1]. In contrast, BioZ and SS-ECG measure electrical potentials. BioZ is an active sensor that requires injecting a low-frequency current and then measuring the resulting change in voltage across a section of tissue. These are converted into changes in impedance as the physical properties of the tissue are altered due to interactions with vasculature, which contracts and expands because of blood flow [8]. In contrast, SS-ECG is a passive sensor that measures the electrical impulse of the heart from a single side of the heart rather than across multiple leads, as is traditionally performed in ECG measurements [9].

Breathing rate (BR) can be estimated from cardiac signals by examining changes in the baseline of the cardiac signal (baseline wander; BW), its AC amplitude (amplitude modulation; AM), and the inter-beat interval (IBI) (frequency modulation; FM). For all signals, FM originates from the process of Respiratory Sinus Arrhythmia (RSA) [7], which leads to increases in instantaneous heart rate during inhalation and the corresponding decreases during exhalation [10]. BW results from changes in the blood circulation on both the venous and arterial sides, which has been theorized to result from changes in intrathoracic pressure or to be mediated by the sympathetic nervous system [11,12]. It has been theorized that AM within PPG and BioZ signals is the result of a decrease in stroke volume, which results in a reduction in cardiac output and reduces the AC amplitude during inhalation and the opposite during exhalation [11]. For SS-ECG, AM results from a rotation of the cardiac electrical vector due to displacements of the heart with each inhalation and exhalation [13]. In previous work, Bao et al. [13] extracted BW from ECG by treating it as an artifact due to body movements with each breath that caused the device or electrodes to shift. In our study, however, we did not observe BW in our SS-ECG measurements in the upper arm, so we did not include BW in the ECG to estimate BR but did include BW for the PPG and BioZ signals.

Our rationale for developing multimodal estimates of HR and BR is that the sensitivity to various noise sources is modality-dependent. For example, PPG is sensitive to differences in skin tones [14], whereas BioZ or ECG are not. In contrast, BioZ and ECG signals are sensitive to power line noise and electromyographic (EMG) noise [15], but PPG is not. Finally, the three modalities are sensitive to the electrode–skin interface in the form of pressure artifacts for PPG and conductivity for ECG and BioZ. Therefore, we hypothesized that combining multiple sensing modalities with different noise sensitivity characteristics would enhance the robustness and/or accuracy of vital sign measurements.

Though several devices have been developed to measure SS-ECG [16,17,18], PPG [19], PPG + SS-ECG [20,21], and BioZ [22] on the upper arm, the proposed device is, to our knowledge, the first to combine PPG, BioZ, and SS-ECG into a wireless wearable device [23]. Our rationale for designing an upper arm wearable device, as opposed to conventional wrist-based systems, is three-fold. First, placing ECG electrodes higher up in the arm, and therefore closer to the heart, increases signal-to-noise ratio (SNR) [24]. Second, prior work [25] has shown that the effect of motion artifacts on PPG due to arm movements are lower in the upper arm than on the wrist. Lastly, our prior work [26] has shown that estimates of HR at the upper arm are more robust to micro-motions than those at the wrist for the three modalities.

Within this study, we assessed the robustness and accuracy of a wireless multimodal upper armband by evaluating its ability to measure HR and BR during a series of selected tasks which induce certain forms of noise. We assessed the trade-off between accuracy and robustness for single-modality and multiple-modality predictions. This study showcases the need to consider a multimodal approach to overcome the limitations of single-modality approaches.

## 2. Materials and Methods

### 2.1. Study Protocol

To investigate the effects of noise on signal quality and HR/BR calculations, we recruited 16 subjects (7 females, 9 males; age range 19–42 years) to complete a study protocol containing several activities that elicited each noise category. Participants provided informed written consent under IRB2022-0227D (Texas A&M University Institutional Review Board). In the study, subjects completed a series of six tasks: sitting sedentary, controlled breathing at a rate of six and ten breaths per minute (BRpm), flexing the arm on which the device was worn (flex and hold for three seconds; relax for three seconds, repeatedly), typing, and texting. Each task lasted two minutes except for controlled breathing (one minute for each breathing rate). A transition period was allowed between each activity and was excluded from the analysis. To assist participants in maintaining their target breathing rate, they followed an audiovisual pacing signal from a smartphone app (Breathing Zone) that showed them when to breathe in and breathe out. We used the sedentary, flexing, typing, and texting tasks to estimate HR, and the controlled breathing task to estimate BR.

Each activity was intentionally selected to introduce different noise sources on signal quality. We examined the effect of skin tone during the sedentary period, the effect of muscle contraction during the arm flexion task, and the effect of external noise during flexing, texting, and typing tasks. Other sources of noise were analyzed across tasks, including human–device noise pertaining to the electrode–skin interface, device placement, and hardware noise from EMI and crosstalk.

To examine the effect of skin tone on signal quality, we grouped participants into three categories based on the Monk Skin Tone (MST) scale [27]: light (MST 1–3) with eight subjects, medium (MST 4–6) with four subjects, and dark (MST 7–10) with four subjects. To obtain ground truth for HR, subjects also wore a Polar H10 (Polar Electro Oy; Kemple, Finland) chest strap that measured ECG in the chest at 130 Hz sampling rate.

### 2.2. Design of the Wearable Device

**Figure 1:**
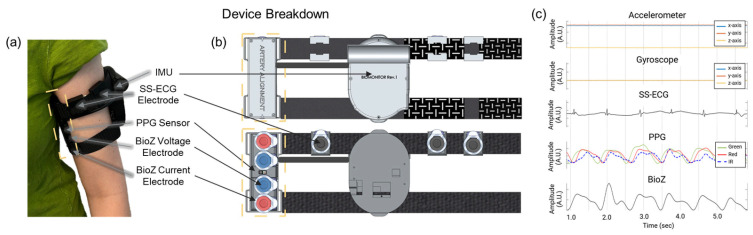
(**a**) Wireless multimodal wearable as worn on the upper arm by a subject, highlighting the artery alignment portion of the device (in yellow). (**b**) Location of the four gel electrodes; the two outer electrodes (red) are for BioZ current injection, and the two inner electrodes (blue) are for the corresponding voltage measurement. The multiwavelength (green, red, IR) PPG sensor is placed between the BioZ electrodes. Three additional gel electrodes (black) are wrapped around the top of the device to measure SS-ECG. (**c**) Sampled signals from the upper arm wearable. Created in BioRender. Branan, K.L. (2024) https://www.biorender.com/.

We designed the wireless sensing device as an armband to be worn on the left upper arm, which is closest to the heart (Figure 1a). The wearable device contains four modalities: (1) multiwavelength PPG (MAX30101—Analog Devices; Wilmington, MA, USA), (2) SS-ECG (AD8232—Analog Devices; Wilmington, MA, USA), (3) BioZ (MAX30009—Analog Devices; Wilmington, MA, USA), and (4) IMU (SM330DHCX—STMicroelectronics; Coppell, TX, USA) containing a 3-axis accelerometer and gyroscope. Careful consideration was given to the placement of all the modalities. The PPG and BioZ sensor/electrodes were placed on the inner portion of the upper arm along the brachial artery. The three electrodes for SS-ECG were placed orthogonal to the line of sensors along the brachial artery and as high up on the arm as possible; see Figure 1b.

To obtain a strong PPG signal, we set the brightness of each LED (green, red, infrared) to its maximum. We used Kendall Ag/AgCl gel electrodes (Cardinal Health; Dublin, OH, USA) to inject AC current for BioZ (65 kHz; 340 µA), measure BioZ voltage signals, and acquire SS-ECG signals. We set the sampling rate for all signals to 100 Hz. The device also contains a microcontroller unit (MCU) (NucleoWB55RG; STMicroelectronics; Coppell, TX, USA) that samples and stores raw data onto an SD card as well as peripheral circuitry (i.e., battery charging, power management, and an external analog-to-digital converter).

### 2.3. Signal Processing Pipeline for Heart and Breathing Rate Estimation

**Figure 2:**
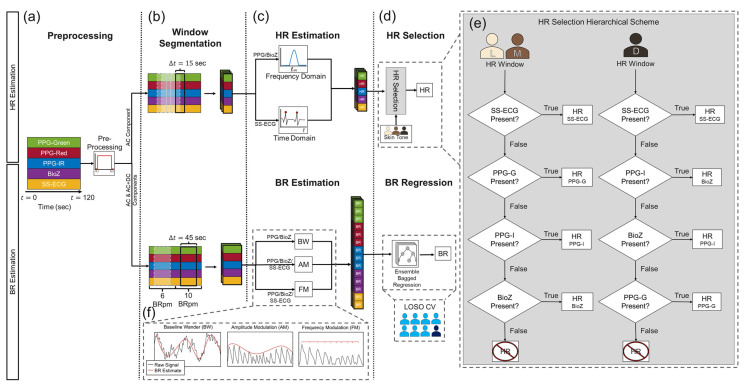
The signal processing pipeline consists of four steps: (**a**) preprocessing, which consists of modality-specific filtering, (**b**) window segmentation, and (**c**) HR/BR estimation techniques leading to (**d**) the HR selection and BR regression algorithms. (**e**) The overall HR estimate for a window was selected based on a modality hierarchical scheme and the skin tone of the subject. BR was computed using a bagged tree ensemble regression model, which was analyzed in a leave-one-subject-out (LOSO) fashion. (**f**) BRs fed into the regression model were computed using three methods: baseline wander (BW), amplitude modulation (AM), and frequency modulation (FM). BW for SS-ECG was not included in the BR estimates.

As shown in Figure 2, we designed a signal processing pipeline consisting of four steps: preprocessing, window segmentation, HR/BR estimation techniques for each sensing modality, and sensor fusion. All signal processing was performed in MATLAB 2024b using built-in functions. For each task, we excluded the first and last 15 s of data from analysis since they contained artifacts due to transitioning to the previous/next task leaving a minute and half of usable signal.

#### 2.3.1. Preprocessing

**Figure 3:**
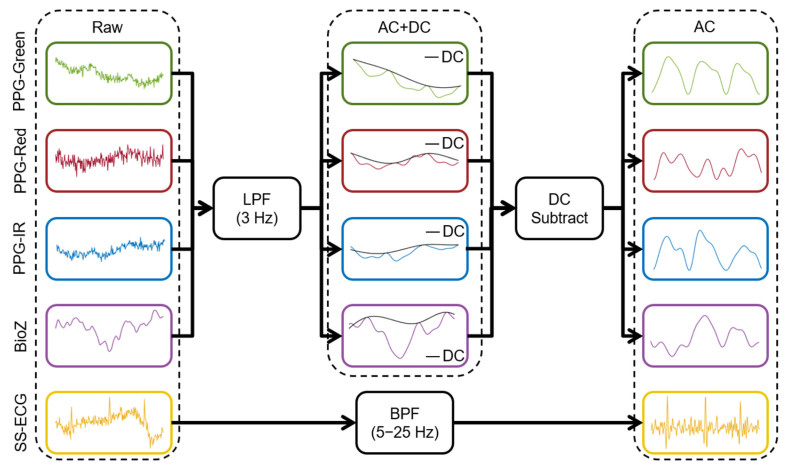
Preprocessing pipeline for all upper arm signals including AC + DC and AC component isolation steps to extract HR and BR.

We used dedicated preprocessing for each sensing modality as seen in Figure 3. For PPG/BioZ, preprocessing consisted of (1) signal detrending (MATLAB ‘detrend’ function), (2) low-pass filtering (LPF) using a fifth-order zero-phase Butterworth with a 3 Hz cutoff frequency (this step yields the AC + DC component), (3) extracting the upper envelope of the signal, (4) subtracting the upper envelope from the AC + DC component, and (5) inverting the resulting signal (to yield the AC component). For SS-ECG and the chest (reference) ECG, preprocessing consisted of (1) signal detrending (MATLAB ‘detrend’ function), and (2) filtering using a fifth-order zero-phase Butterworth bandpass filter (BPF) with 5–25 Hz cutoffs for SS-ECG and 0.5–30 Hz cutoffs for chest ECG. The AC component of each signal was used for HR estimations while the AC and AC + DC components were used for BR estimations.

#### 2.3.2. Window Segmentation

- To estimate HR, we used a 15 s sliding window with a 50% overlap for the sedentary, flexing, typing, and texting tasks. To estimate BRs during the controlled breathing task, we split the 90 s controlled breathing trial into two 45 s windows representing the breathing rates 6 and 10 BRpm.

#### 2.3.3. Estimation of Heart Rate

- We estimated HR from each 15 s window for each of the sensor signals. For SS-ECG and chest ECG, we isolated the R-peaks and extracted the average IBI to compute HR. For PPG/BioZ HR, we computed the power spectral density (PSD) of the signal, isolated the main frequency peak in the range of 0.67–2 Hz, and converted to HR. For both approaches, we eliminated HR estimates less than 40 bpm and greater than 120 bpm as the subjects were not performing extraneous activity and a typical resting HR ranges from 60 to 100 bpm [28]. If an estimate was outside the range of 40–120 bpm, the estimate was discarded.

#### 2.3.4. Estimation of Breathing Rate

To estimate BR, we extracted the BW component from PPG and BioZ, and the AM and FM components from each of the three modalities:

- To extract BW, we extracted the upper and lower envelopes of the AC + DC component using a peak-finding envelope filter averaged them, smoothed the data with a Gaussian smoothing filter, and extracted the peak frequency from the PSD of the corresponding signal, and converted it into BR; see left column in Figure 2f.
- To extract the AM component, we extracted the upper envelope of the AC component, followed by detrending and smoothing; see middle column in Figure 2f. Then, we computed the PSD, extracted the main frequency, and converted it into BR.
- To extract the FM component, we first computed the IBI time series (R-peaks for SS-ECG; systolic-peaks for PPG/BioZ) of the AC component; see right column in Figure 2f. Then, we detrended the time series interpolated the time series to a higher sampling rate and smoothed the time series with a gaussian smoothing filter. We computed the PSD, isolated the main frequency and converted it into BR.

#### 2.3.5. Multimodal Prediction of Heart and Breathing Rates

To estimate HR, we developed a hierarchical scheme based on the which sensing modalities supplied valid HRs (HR estimates within the predefined threshold) and the subject’s skin tone that resulted in five independent HR estimates (three PPG wavelengths in the frequency domain, one BioZ in the frequency domain, one SS-ECG in the time domain) and 14 independent BR estimates: BW from three PPG + BioZ and AM and FM from three PPG + BioZ + SS-ECG (Figure 2c).

To obtain a single HR prediction from the five HR estimates, we used a hierarchical scheme, as described in the flowchart of Figure 2d. Namely, if a valid HR prediction (i.e., within limits) was available from SS-ECG, we used that prediction and ignored predictions from the remaining modalities; otherwise, we used the HR prediction from the PPG channel that was most robust for the participant’s skin tone or BioZ (if available): (1) for dark skin tone, we reported predictions from PPG-IR, BioZ, or PPG-green, in that order; (2) for light and medium skin tone, we used the following order: PPG-green, PPG-IR or BioZ. The effects melanin has on light absorption were considered when deciding the order of the hierarchical scheme. This is due to melanin absorbing green light more than IR and because dark skin tone individuals have higher epidermal melanin concentrations, leading to a decrease in SNR for PPG-green signals relative to those collected from light/medium skin tone individuals. Hence, PPG-IR appeared earlier in the scheme for darker skin tone individuals compared to light/medium skin tones as PPG-IR has a higher SNR relative to other wavelengths for these skin tone levels. We report prediction accuracy in terms of the mean absolute error (MAE) and standard deviation of each individual modality.

To obtain a single BR prediction from the 14 BR estimates, we used the Regression Learner App to train a robust linear regression model, which uses iteratively reweighted least squares to compute the regression coefficients [29]. The selected hyperparameters for our ensemble bagged decision tree regression model included a minimum leaf size of 4, 136 learners, and used 13 predictors to sample. During training, k-folds cross validation (CV), where k = 5, is applied to prevent overfitting and evaluate the model performance on the training set. The model is analyzed in a leave-one-subject-out (LOSO) fashion. As with HR, we report MAE and standard deviation (on the test subject) of each individual modality, plus those from the robust linear fit and the estimated feature importance that the model returns.

## 3. Results

**Figure 4:**
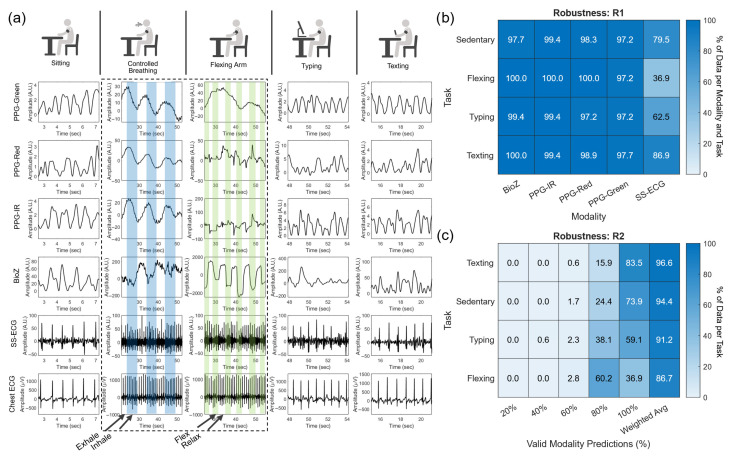
(**a**) Sample signals from each cardiac sensing modality (row) and each task (column) for a subject in the study. Blue shading in the controlled breathing task denotes the inhale phase. Green shading in the arm-flexing task represents moments of flexing. Distribution of HR estimate windows (**b**) for each task (e.g., sedentary, flexing, typing, and texting) and modality and (**c**) across each task containing an HR estimate from 100%, 80%, 60%, 40%, and 20% of the modalities.

Figure 4 illustrates the signals obtained from each modality for each of the five tasks for one of the subjects in the study. We show the AC signal for all but the controlled breathing and arm flexion tasks, for which the dominant effect is on the quasi-DC offset. The PPG and BioZ signals show a distinct BW component during the breathing task. PPG has a lower DC offset during the inhale phase and a higher DC offset during the exhale phase; the opposite trend can be seen in the BioZ signal. In contrast, the ECG signal only shows an FM component (longer IBIs during exhalation). The flexing task involves muscle contractions that introduce EMG noise, as seen by a characteristic square waveform in the BW of BioZ with a lower DC offset during flexing. In contrast, the SS-ECG signal exhibits higher background noise during flexing due to the electrode not maintaining complete contact with the skin. Flexing also introduces significant motion artifacts on PPG, most noticeably on the red and IR channels, as muscular motions change the density of tissue. Finally, typing and texting tasks introduce noise with similar morphology and frequency as those on the sedentary signals, except for BioZ, which is more sensitive than PPG and SS-ECG to subtle muscular movements.

### 3.1. Distribution of Heart Rate Estimations

The window segmentation procedure results in 11 analysis windows for each task and modality. To measure robustness, we computed the percentage of analysis windows with valid HR estimates per modality (robustness measure R1), and the percentage of sensing modalities with valid HR estimates per analysis window (R2). Results are shown in Figure 4b (R1) and Figure 4c (R2). The R1 robustness of the three PPG channels and the BioZ channel is very high for all tasks (R1>97%) but varies significantly across tasks for the SS-ECG channel (37%<R1<80%). The latter is likely due to muscular contraction during flexing, shifting the SS-ECG electrodes or introducing EMG noise, which resulted in the inability to utilize SS-ECG for HR estimation. When examining R2 robustness, most of the analysis windows have valid HR estimates for 4–5 sensing modalities regardless of task (80%<R2<100%), but R2 drops for tasks that inherently lead to more motion artifacts, as one may expect. For example, the highest weighted average for R2 is for the texting task (97%) and the lowest is for the flexing task (87%). From here on, we will not refer to each individual task but rather aggregate all the data to accurately predict HR during various noise conditions.

### 3.2. Effects of Skin Tone on Heart Rate Estimation

**Figure 5:**
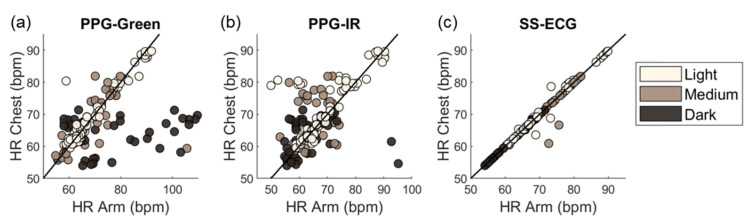
Deviation in HR predictions relative to the chest reference HR in terms of skin tone and modality: PPG-green (**a**), PPG-IR (**b**), and SS-ECG (**c**).

A significant source of between-subjects noise in PPG signals is skin tone due to the higher concentration of melanin in the epidermis for darker skin tones. The effect is more pronounced for the green channel because melanin absorbs green wavelengths more strongly than red and IR wavelengths. Figure 5 shows HR predictions vs. ground truth for PPG-green and compares against those from PPG-IR (which is less affected by skin tone) and SS-ECG (which is not affected). HR estimates for PPG-green show larger deviations from ground truth for dark and medium skin tones than for light skin tones.

### 3.3. Unimodal and Multimodal Estimation of Heart Rate

**Figure 6:**
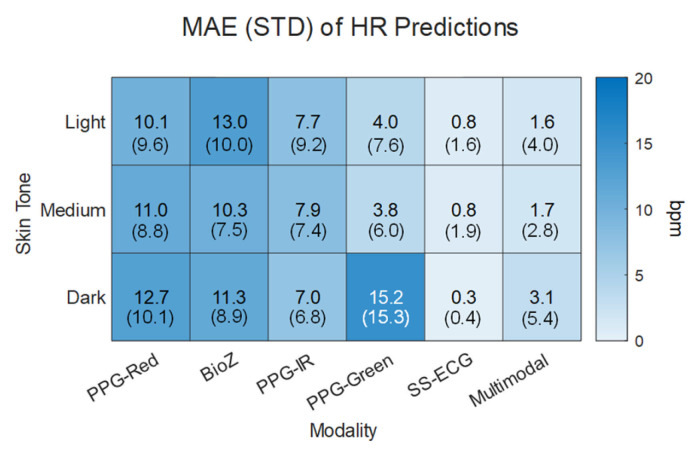
Mean absolute error (MAE) and standard deviation (STD) of the absolute error for unimodal predictions and multimodal prediction in terms of the modality and skin tone.

We compared HR estimates from each individual modality against those based on the multimodal hierarchical scheme. Results are summarized in Figure 6 as a function of skin tones. SS-ECG and the multimodal approach had the lowest overall MAE (0.6 ± 1.3 bpm and 2.1 ± 4.1 bpm) for all skin tones. PPG-red and BioZ had the largest overall MAEs. When considering individual modality HR predictions per skin tone, PPG-green provided the lowest MAE, outside of SS-ECG, for light and medium skin tones while the MAE dark skin tone individuals yielded the largest MAE for any individual modality prediction.

### 3.4. Unimodal and Multimodal Estimation of Breathing Rate

We compared BR estimates from each of the five sensing modalities using the three extraction approaches (BW, AM, FM), except for SS-ECG, which does not exhibit BW. Results are shown in Table 1. When considering unimodal predictions from AM, SS-ECG has the lowest MAE and BioZ the highest. Similar results are observed for unimodal predictions from FM. Interestingly, unimodal predictions from baseline wander show the opposite result, with BioZ having the lowest MAE and PPG-green the highest, which suggests that BW predictions are somewhat orthogonal to those from AM and FM. Most importantly, when we combine predictions from the 14 channels using an ensemble bagged tree regression model, we achieve an MAE that is lower than best individual modality (BioZ-BW). Also, interestingly, when combining predictions from all methods for BioZ, we achieve the lowest MAE relative to predictions across all channels.

In the final step, we analyzed the feature importance of the ensemble bagged tree regression model when utilizing all 14 channels as predictions. Surprisingly, the most important of all 14 features are BW for BioZ and FM for SS-ECG, with BW for BioZ making up at least 80–95% of the importance for all splits where splits were based on a leave-one-subject-out (LOSO) analysis technique. FM for SS-ECG comprised 5–20% of the importance score. Thus, despite BioZ having a high MAE for HR estimations (±10 bpm with respect to reference), its information plays a critical role when estimating BR, further illustrating its complementarity for cardiorespiratory monitoring.

## 4. Discussion

Several noise-targeting tasks were performed to understand and test how the noise introduces artifacts within the sampled biosignals, causing inaccuracies in HR estimations. The tasks were selected to include the four noise categories primarily for heart rate estimations: physiological, external, hardware, and human–device. In addition, respiration effects were analyzed for two controlled breathing rates, 6 and 10 BRpm. A summary of the effects of these noise sources on the PPG, BioZ, and SS-ECG signals obtained from this study can be seen in Table 2. In addition, further details related to these results can be found in the proceeding discussion.

### 4.1. Physiological Noise: Skin Tone and Muscular Activity

The physiological noise sources included skin tone and muscular contraction. Skin tone effects on signal quality were seen during the sedentary period and muscular contraction noise effects were analyzed during flexing and typing as typing also requires micro arm movements. When considering skin tone effects, melanin absorbs green light more than IR, causing a lower SNR for shorter wavelength PPG signals when measuring darker skin tone individuals [30]. As seen within our study, a lower MAE was achieved for IR HR estimations compared to green for dark skin tone individuals. However, according to several studies, skin tone variation or an increase in the amount of epidermal melanin does not play a significant effect on the HR accuracy for PPG-green [31,32]. It is crucial to note that there is no consensus on whether skin tone plays a significant role in the ability to accurately estimate HR since there are mixed results that are highly dependent on the design (e.g., source-detector separation, wavelengths, reflectance mode, transmission mode) and physiological location of the device used (e.g., wrist, arm, chest, or finger).

In terms of muscular activity, both BioZ and SS-ECG are highly affected by EMG or muscular contraction noise [3], resulting in less usable data. Lázaro et al. found that the best channel of SS-ECG produced a median of usable data segments of 45.02% for daytime measurements relative to 99.06% during nighttime, showing the effects of EMG on the amount of usable signal [17]. Both noise sources occur in activities such as flexing and typing and only 66% of the SS-ECG data in our study was considered usable (defined as HR estimates that were within the limits). EMG could also result in poor electrode–skin interface noise due to the physical motion of the muscles on the device.

### 4.2. External Noise: Motion Artifacts

External noise sources, such as motion, were investigated through the typing and texting tasks. As mentioned, motion causes EMG noise along with device movement which results in a lack of electrode adherence as seen during the typing task for SS-ECG. Overall, BioZ provided poor HR estimations (MAE > 10 bpm), which can be attributed to the artifacts introduced by motion. When comparing how motion affects PPG signals, there was a significant amount of usable data during these tasks (i.e., >97%); however, there was general lack of accuracy for all PPG signals (i.e., MAE > ~4.0 bpm), despite skin tone, attributed to potential motion artifacts within this study. Other studies also compared green and IR PPG and found that motion causes a larger fluctuation in signal amplitude for IR compared to green when measuring on the upper arm [25] which, in theory, could cause a larger discrepancy in HR estimations. Lee et al. also found that longer wavelengths were affected more in terms of SNR and HR estimations during smaller motions when measuring signals on the finger [33]. It is important to also note that skin tone was not reported for either of these studies and could play a role in the discrepancies within their results.

### 4.3. Hardware and Human–Device Noise

Hardware noise, such as electromagnetic interference, was present but easily negated with digital filtering during the preprocessing segment of the data analysis pipeline (Figure 2). Crosstalk between the sensors was not detected during testing; however, it is important to mention that one reason for selecting an injection frequency of 65 kHz for the BioZ current signal was, in part, because anything higher would introduce crosstalk into the SS-ECG system, saturating the biosignal.

Human–device noise, including device location and the electrode–skin interface, was also investigated during flexing, typing, and texting tasks, as the movement of the arm could cause the electrodes to lose contact with the arm or cause the device to migrate, causing a fluctuation in the collected biosignals. For most noise-inducing tasks, SS-ECG outperformed PPG and BioZ regarding the resulting average MAE for HR estimations (Figure 6). This is due to the modality’s SNR, which can be partially attributed to the electrodes used, as the gel electrodes adhere to the skin maintaining reasonably good contact during experimental tasks. However, there are instances in which the electrodes partially or completely pop off the skin, introducing noise and quickly saturating the small magnitude SS-ECG signal (i.e., flexing) resulting in less useable data from the modality and therefore we found a trade-off between accuracy and robustness when utilizing SS-ECG.

### 4.4. Comparing PPG Wavelengths

When comparing the results obtained from the multiwavelength PPG sensors, we noticed that PPG-green had the lowest MAE for all the estimates from all available windows without the distinction of skin tone, whereas PPG-red had the highest MAE. There are several theories on the discrepancies in HR estimations between the wavelengths. One theory is based on the penetration depth of each wavelength. When comparing green and red wavelengths, red is the longer wavelength and, therefore, penetrates deeper, increasing the noise introduced in the collected signal [34]. When comparing the discrepancies between both of the longer wavelengths (PPG-red and PPG-IR), dependent on the exact wavelengths of red and IR light used (i.e., 660 and 880 nm), the absorption of light by the dominant chromophore within the blood, oxyhemoglobin, for a highly oxygenated subjects (as was the case in this study for all subjects), is almost an order of magnitude larger for IR light compared to red. Water absorption is nearly flat in this wavelength range while absorption from melanin monotonically decreases with increasing wavelength. IR light absorbs melanin almost an order of magnitude less compared to red [35]. The absorption coefficients of all three chromophores in the red region can contribute to the decreased SNR of PPG-red resulting in a higher MAE for HR estimations for all skin tones. However, it is important to note that deoxyhemoglobin absorbs red light more than IR and so for non-healthy subjects, whose blood oxygenation is compromised, deoxyhemoglobin is higher, and thus, the red wavelength may have more utility. The location of the system such as the forearm [36] and upper arm [25] also have a lower signal amplitude compared to other locations, such as the finger, wrist, or forehead, due to decreased blood volume within the probed tissue. Because of this, noise artifacts could saturate the measured PPG signals.

### 4.5. Bioimpedance Signal Limitations and Utility

The average MAE for HR estimations based on BioZ was, in general, the highest for all modalities, except for the PPG-green on dark skin tones and the PPG-red on medium/dark skin tones. One reason for this can be the placement of electrodes. If the device is not perfectly aligned with the artery, the signal quality can degrade. The brachial artery is deeper relative to other locations where BioZ is measured (e.g., radial artery on the wrist), and therefore, the injection frequency may need to be tuned to each subject, which was not performed in this study. Another reason for higher MAEs is the composition of each subject’s arm. There is a larger tissue volume on the upper arm containing more muscle and fat which could cause discrepancies in the acquired BioZ signal.

### 4.6. Breathing Rate Estimation

Despite the poor performance of BioZ for HR estimations, extracting the BW from the BioZ signal to estimate BR produced the lowest MAE compared to all other modalities and estimation techniques (Table 1). For almost all PPG and BioZ estimates, BW had the lowest MAE which follows similar trends from Charleton et al., where BW approaches produced the lowest MAE results compared to FM and AM analysis [37]. Overall, AM produced the highest MAE for almost all modalities. This is likely due to the lower SNR of all measurement techniques on the upper arm; therefore, definitive AM breathing frequency could not be identified. Fusing results from each BR estimation method using a bagged tree ensemble regression model showed that the predominate method for BR estimation was BW on BioZ. The overall MAE was lowered when fusing results across multiple BR extraction techniques instead of a single technique (Table 1). This aligned with Charleton et al. and Birrenkott et al. where both groups considered fusing results from BW, AM, and FM from PPG and ECG predictions decreasing the MAE compared to individual modality and technique predictions [37,38].

### 4.7. Multimodal Fusion Improves Robustness

Our work showed that single-modality HR predictions are not as robust as fusing results from multiple modalities due to the targeted noise sources by which each modality is affected. Using multimodal fusion for both HR and BR estimations allowed for the use of more predictions to be analyzed and an improvement in MAE. Since noise sources affect modalities differently (i.e., EMG affecting SS-ECG and not PPG), the use of multiple modalities helps to overcome the limitations of a single-modality prediction. At least 95% of the PPG and BioZ data in our study was usable but did not provide as low of an MAE when evaluated unimodally. Although SS-ECG had the lowest MAE, only 66% of the data was usable. This showed the trade-off of accuracy versus robustness in SS-ECG. By applying the hierarchical scheme, we were able to predict HR from all the analysis windows and overcome each of the sensors’ limitations, creating a more robust and accurate continuous system.

### 4.8. Limitations

This study has its limitations. In particular, the dynamic range for HR and BR was small since subjects performed tasks that did not include elevating their HR and were tasked with only breathing at two designated rates. Also, the sampled population consisted of 16 subjects, creating a lack of generalizability since the sample size was small.

## 5. Conclusions

The combination of multiple modalities (multiwavelength PPG, BioZ, and SS-ECG) within a novel, wireless, upper armband wearable device with a hierarchical scheme for HR estimation and a regression model for BR estimation, in general, improved performance relative to a single-modality method. Overall, the use of multiple-modality wearable devices to overcome noise sources that are specific to individual modalities (i.e., skin tone for PPG and EMG for SS-ECG) provides a better overall accuracy and robustness.

## Figures and Tables

**Table 1:** MAE and standard deviation of the error for each modality and BR estimation method and for the regression approach.

Method	Red	IR	Green	BioZ	SS-ECG	Regression (14-Channels)
AM	4.5 (3.4)	3.7 (3.6)	2.4 (2.3)	4.7 (3.7)	2.3 (3.9)	-
FM	4.0 (3.1)	3.3 (2.8)	3.7 (3.8)	4.4 (3.9)	0.97 (1.2)	-
BW	2.1 (2.3)	1.9 (2.1)	2.9 (2.4)	0.57 (0.80)	-	-
Regression	0.97 (0.62)	1.5 (0.85)	1.7 (0.74)	0.13 (0.27)	0.66 (0.88)	0.22 (0.37)

**Table 2:** Summary of the noise sensitivity by modality for HR predictions.

Noise Category	Noise Source	Modality				
		PPG-Green	PPG-Red	PPG-IR	BioZ	SS-ECG
Physiological	Skin Tone	High	Moderate	Low	None	None
	EMG (Flexing)	None	None	None	High	High
External	Motion (Typing)	Low	Moderate	Low	High	Moderate
	Motion (Texting)	Low	Moderate	Low	High	Low
Hardware	Crosstalk	None	None	None	None	None
	EMI	None	None	None	None	None
Human–Device	Electrode–Skin Interface	None	None	None	High	High
	Location	Low	Low	Low	High	High

Low: SNR ≥ 3 or robustness (R1) ≥ 90%. Moderate: 3 > SNR ≥ 1 or 90% > robustness (R1) ≥ 50%. High: SNR < 1 or robustness (R1) < 50% or MAE > 10 bpm (criteria for skin tone noise only). None: not observed.

## Data Availability

The raw data supporting the conclusions of this article will be made available by the authors on request.
